# Morphofunctional characteristics of flight-related traits in deltamethrin-resistant and susceptible *Triatoma infestans* (Klug, 1834) of the Argentinean Chaco

**DOI:** 10.1186/s13071-025-06678-2

**Published:** 2025-03-06

**Authors:** Víctor A. Maza, M. Victoria Cardinal, Julieta Nattero

**Affiliations:** 1 Laboratorio de Eco-Epidemiología, DEGE (FCEN, UBA), IEGEBA (UBA/CONICET), Intendente Güiraldes 2160-Ciudad Universitaria - Pabellón 2, Ciudad Autónoma de Buenos Aires, CP: C1428EGA Argentina; 2 Departamento de Ecología Genética y Evolución, Laboratorio de Eco-Epidemiología, Facultad de Ciencias Exactas y Naturales, Universidad de Buenos Aires, Ciudad Autónoma de Buenos Aires, Argentina; 3 Departamento de Biodiversidad y Biología Experimental. Facultad de Ciencias Exactas y Naturales, Universidad de Buenos Aires, Ciudad Autónoma de Buenos Aires, Argentina

**Keywords:** Morphometric traits, Pyrethroid resistance, Hemelytra, Stiff and membranous parts of the forewing, Toxicological groups, Flight-related traits, Flight muscle development

## Abstract

**Background:**

Chagas disease, transmitted by triatomine bugs, is a major vector-borne parasitic disease in Latin America. *Triatoma infestans*, the principal vector in the Southern Cone, is primarily controlled through residual insecticide spraying. However, resistance to pyrethroids, especially in Northern Argentina and Southern Bolivia, has emerged. Resistant *T. infestans* populations exhibit reduced fitness, including impacts on reproductive success and dispersal capacity. This study investigates the flight potential and morphological changes in *T. infestans* populations with varying levels of insecticide resistance, hypothesizing that resistance may induce morphological changes in wing and head structures related to dispersal.

**Methods:**

We analyzed three resistance profiles of *T. infestans*—susceptible (S), moderately resistant (MR), and highly resistant (HR)—collected from ten domestic or peridomestic sites in two municipalities from Chaco province, Argentina. We registered flight muscle development and measured flight-related traits (wings, heads, and the stiff and membranous portions of the wing) using a landmark-based methodology. We also assessed morphological disparity and covariation of these traits across toxicological groups.

**Results:**

Significant morphological differences were found between resistant and susceptible populations. The frequency of insects with and without muscle varied across toxicological groups only for females, exhibiting the highest proportion of HR insects with fight muscle (86.21%). MR and HR males exhibited smaller stiff portions of the wing and heads than S males. Shape variation analysis showed that S females had wider forewings than resistant females, while HR females had narrower wings with a wider stiff portion. Susceptible males had wider and longer wings compared with resistant groups. Additionally, resistant populations showed greater morphological disparity and reduced covariation between flight-related traits.

**Conclusions:**

Our study shows that pyrethroid resistance in *T. infestans* is linked to morphological changes in flight-related traits. These changes suggest a tradeoff between resistance and flight capacity, with energy allocated to resistance mechanisms potentially limiting flight. The reduced covariation between flight traits in resistant individuals supports the idea of pleiotropic effects. While resistant individuals may perform better in insecticide treated areas, their reduced flight capacity could limit long-distance dispersal, affecting population dynamics and vector control efforts.

**Graphical abstract:**

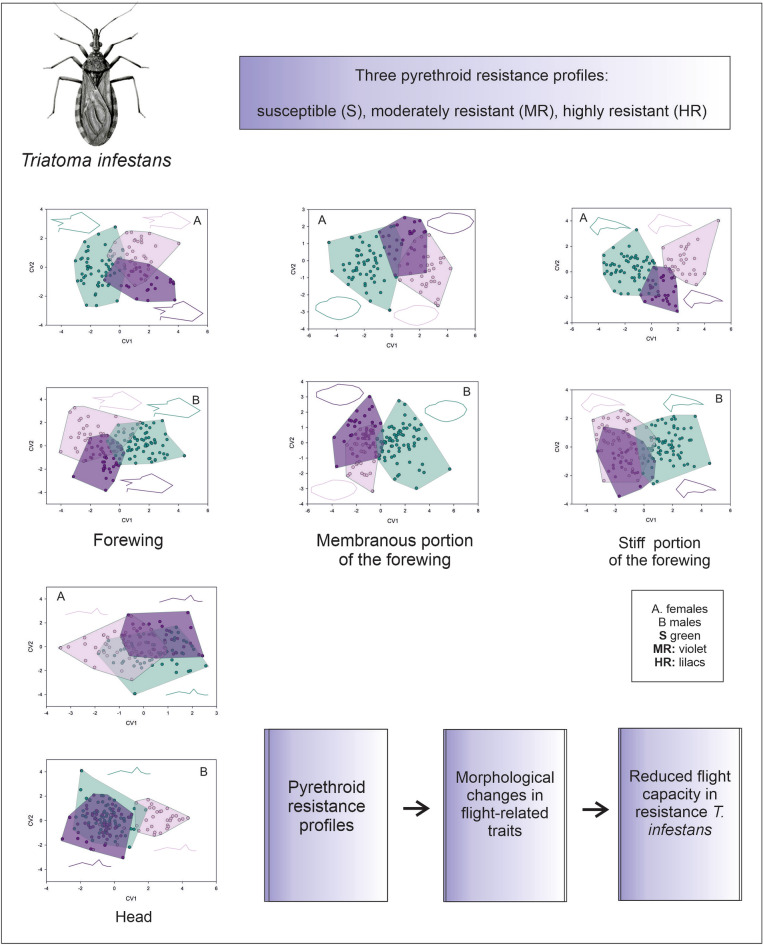

**Supplementary Information:**

The online version contains supplementary material available at 10.1186/s13071-025-06678-2.

## Background

Chagas disease remains the most significant vector-borne parasitic disease in Latin America, primarily transmitted by various species of triatomine bugs [1]. Among these, *Triatoma infestans* (Klug, 1834) serves as the main vector in the Southern Cone of South America [2]. The adaptability of these vectors to human habitats and the lack of preventive vaccines have made residual insecticide spraying of houses the primary strategy for preventing new cases. Synthetic pyrethroids are the main class of insecticides employed in the control of triatomines [3]. Although pyrethroids have been initially effective in managing vector populations, resistance has emerged, paralleling trends observed in other pest species treated with these chemicals [4, 5].

Resistance to pyrethroids was first documented as a significant issue in northern Argentina and southern Bolivia in the early 2000s [6]. While these areas were identified as hotspots, subsequent reports have revealed ongoing challenges in vector control associated with pyrethroid-resistant populations of *T. infestans* [7, 8]. Recent studies have identified new resistance hotspots in two departments of Chaco Province (Güemes and Independencia), Argentina [9, 10]. The Güemes department exhibits a toxicological profile characterized by the highest levels of deltamethrin resistance [9]. Furthermore, a recent investigation has linked environmental variables to the observed toxicological heterogeneity in this region [11]. In contrast, the Independencia department shows incipient-to-moderate pyrethroid resistance, particularly along a rural-to-urban gradient [10].

The adaptive costs associated with insect-resistant populations in environments devoid of insecticides are often explained through pleiotropic effects [12, 13]. Viewing the development of resistance as a microevolutionary process, where natural selection is a driving force [3, 14], allows for a better understanding of the tradeoffs among phenotypic and physiological traits. These traits can confer adaptive advantages, present negative consequences for resistant individuals in insecticide environments, or remain neutral [15]. The resistant phenotype arises from two primary physiological and biochemical processes triggered by insecticide exposure, as well as changes in detoxification mechanisms that contribute to resistance [3]. As noted, genetic background plays a crucial role in these resistant populations; alterations in these processes can affect other phenotypic traits or reduce energetic investments. Recent studies on resistant *T. infestans* populations have revealed that resistant individuals often experience reduced fitness, as evidenced by prolonged nymphal stage durations, fewer reproductive days, and lower hatching success [16]. Laboratory investigations have further demonstrated diminished fecundity and fertility, alongside increased nymphal stage durations [17, 18]. Additionally, variations in active dispersal and reproductive output have been observed [19–21], as well as changes in the timing of excretion/defecation and chemical communication [22–24]. Collectively, these findings support the pleiotropic hypothesis regarding *T. infestans* populations, which is increasingly relevant for the development of new vector control strategies, particularly in light of altered vectorial capacity [25].

When two or more structures within an organism serve a common function, their phenotypic relationship is termed morphological integration [26, 27]. This integration is typically assessed by examining the degree of association or covariation between these structures. A strong covariation indicates that the structures are highly integrated, working together to optimize a specific function (e.g., flight). In contrast, weaker covariation may suggest more independent functioning of these traits, potentially reflecting reduced functional synergy [26, 27]. The degree of morphological integration can, therefore, provide insights into the evolutionary constraints and tradeoffs influencing the development of functionally related traits.

While numerous studies have examined morphological changes in triatomines, emphasizing their role as indicators of microevolutionary processes, there is a lack of research specifically addressing the morphological effects of insecticides. For instance, alterations in cuticle thickness, wing size, and wing shape in pyrethroid-resistant populations of *T. infestans* exposed to sublethal doses of deltamethrin were reported [28]. Additionally, evidence of reduced dispersal capacity in pyrethroid-resistant individuals, compared with their susceptible counterparts, highlights phenotypic modifications in the wings, head, and antennae among populations from Chaco Province, Argentina [29]. Flight dispersal is recognized as the primary mechanism for colonization and reinfestation in triatomine populations [30, 31]. The substantial flight potential of *T. infestans* has been highlighted, reinforcing the notion that flight dispersal is critical after insecticide spraying [32]. Reports indicate that the dispersal flight range for *T. infestans* can vary from 200 m to 1500 m [33, 34]. This capacity for dispersal is vital for understanding historical vector prevalence and the dynamics of insecticide resistance. Understanding the constraints and tradeoffs associated with the pyrethroid-resistant phenotype is crucial for predicting reinfestation dynamics and resistance prevalence following insecticide application. The primary aim of this study is to characterize the flight potential of *T. infestans* field populations with differing insecticide resistance profiles. We register the flight muscle development and measure the shape and size of the head and wings, guided by the central hypothesis that resistance may incur in differences in flight muscle development and morphological changes in wing and head structures, which could affect dispersal capacity. We expect to find the greatest differences between highly resistant individuals and susceptible ones.

## Methods

### Study area and insects

Fieldwork was carried out in two municipalities (Avia Terai and Juan José Castelli) of Chaco province, Argentina, as described elsewhere [10, 16, 35]. These localities were located approximately 80 km apart. Adults from *T. infestans* used in the current study were collected during cross-sectional surveys of house infestation with triatomines between February and May 2018 by timed-manual collection. Insects used for this study were collected from ten domestic or peridomestic sites (i.e., three in Juan José Castelli and seven in Avia Terai) (Table 1). Collected *T. infestans* adults were transported to the insectary at the Facultad de Ciencias Exactas y Naturales, Universidad de Buenos Aires (FCEN UBA) and maintained according to the collection sites as free-mating separate stocks with an average room temperature of 24.5 °C ± 3 °C and relative humidity of 56.7% ± 13%. Insects were regularly fed on live chickens, with restricted movement (methodology described in [7]). The emerging I nymphal stage individuals from each collection site were tested for deltamethrin susceptibility at the reference laboratory (Centro de Investigaciones de Plagas e Insecticidas, Villa Martelli, Argentina) following a standardized protocol [36]. This standardized protocol was used for monitoring, and evaluation of pyrethroid resistance allows for comparison of the results obtained by different laboratories.

**Table 1:** Details from the origin of the studied individuals of *Triatoma infestans* from the three toxicological studied groups

Toxicological group	Municipality of origin	Collection habitat	Wings		Head	
			Females	Males	Females	Males
HR	Castelli	Domicile and peridomicile	29	30	44	36
MR	Avia Terai	Peridomicile	29	49	37	56
S	Avia Terai	Domicile and peridomicile	58	73	59	75

*HR* high resistance, *MR* moderate resistance, *S* susceptible

Studying triatomine insecticide resistance. Results from the deltamethrin susceptibility test allow us to define three toxicological groups according to the observed survival [9]; the susceptible group (S) exhibited a survival < 20%, whereas the highly resistant (HR) and moderately resistant (MR) group exhibited a survival > 80% and between 20% and 80%, respectively (Table 1).

### Data collection

**Fig. 1:**
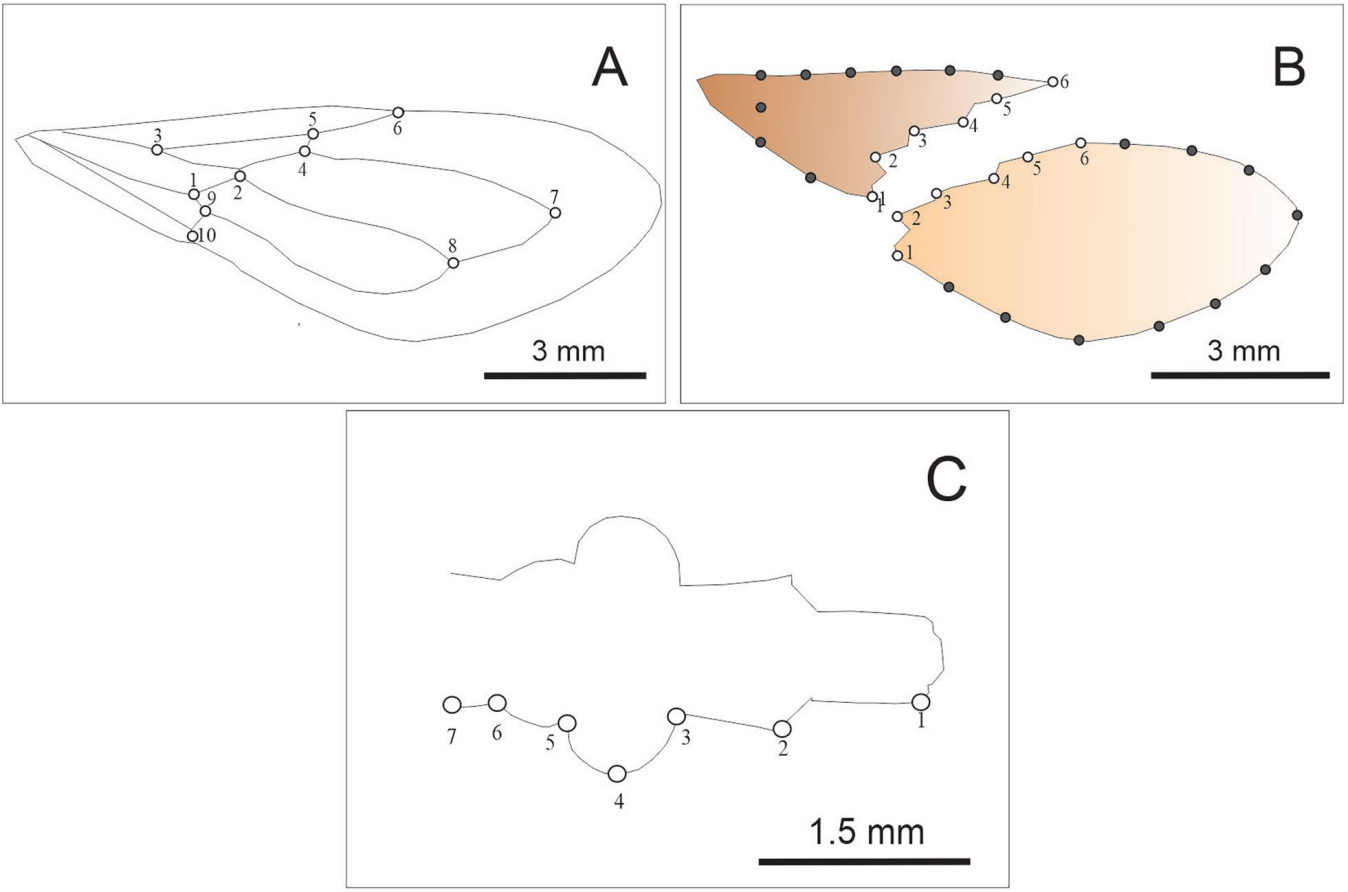
Landmark (open circles) position for the stiff and membranous portions of the forewing (**A**), landmark and semilandmark (grey-filled dots) position in the contour of the stiff (brown) and membranous (light orange) portions of the forewing (**B**), and landmark position for the right side of the head (**C**) for studied *T. garciabesi* individuals

A total of 140 females and 167 males belonging to the three toxicological groups were included in this study (i.e., F0). At the laboratory, frozen females and males were dissected by removing the head and pronotum, and the thoracic cavity, where flight muscles are located, was observed under a stereomicroscope (Zeiss SV11, Germany). The presence or lack of developed muscles was determined for each individual. Digital images of the dorsal view of the right forewing and head were taken using a digital camera (model S9900; Nikon Corp., Tokyo, Japan) mounted on a stereomicroscope (model Stemi SV-11; Carl Zeiss AG, Jena, Germany) at 6 × magnification. All images included a reference scale. For assessing wing size and shape variation across toxicological groups, we used landmark-based geometric morphometry. For the forewing, we collected ten type I landmarks positioned at vein intersections as described elsewhere [37] (Additional file 1) (Fig. 1a). For studying morphometry variation of the contour of membranous and stiff portions of the forewing, a combination of landmarks and semilandmarks were used. Semilandmarks were used to analyze the lateral contour of both the membranous and stiff portion of the forewing, where no sufficient homologous points were detected to characterize the morphology of the specimens using traditional landmarks (Fig. 1b). For the membranous part, six type I landmarks were used to characterize the shape of the boundary between the stiff and membranous parts, and ten equidistant semilandmarks were used to characterize the lateral contours (Additional file 1) (Fig. 1b). For the stiff portion, six type I landmarks were used to characterize the shape of the boundary between the stiff and membranous parts, and nine equidistant semilandmarks were used to characterize the lateral contours of this part (Additional file 1) (Fig. 1b). Semilandmarks were transformed to landmarks and analyzed together with traditional landmarks [38]. For the head, seven coplanar type II landmarks on the right side of the ventral view of the head were defined and collected; these landmarks included the anterocular, ocular, postocular, and neck regions (Additional file 1) (Fig. 1c). Landmark and semilandmark collection was carried out using TPSdig 2.31 software [39]. When a structure or part of a structure was damaged, it was not included. The final number of the structures included for each toxicological group is presented in Table 1. Data on forewing, stiff and membranous portions, and head shape were extracted with a generalized full Procrustes fit and a projection.

### Data analysis

All analyses were done for females and males separately since *T. infestans* was reported as a sexual dimorphic species for morphometric traits (e.g., [29, 40, 41]). Chi-squared tests were used to compare the frequency of females and males with and without flight muscles across toxicological groups using the package *dplyr* of R, running on RStudio (RStudio Inc., Boston, MA, USA). To quantify variation in the forewing, membranous and stiff portions of the forewing, and head size and shape, a quantitative shape analysis was also performed using geometric morphometry on the basis of the statistical analysis of landmark coordinates. We computed centroid size (CS) (i.e., the square root of the sum of squared distances from each landmark to the centroid of the configuration) as a measure of size for each structure or portion of the structures measured [42]. We first compared forewing shape variation for females and males with and without flight muscles for each toxicological group, since a previous study had detected wing shape differences associated with flight muscle dimorphism [43]. Our analysis showed no significant (p > 0.05) Procrustes and Mahalanobis distances in all cases, and morphometric analysis was done without considering the presence or absence of flight muscles. Data on forewing, membranous and stiff portions of the wings, and head were extracted with a generalized full Procrustes fit and a projection to shape tangent space and Procrustes coordinates, as shape variables were used. To analyze differences in shape configuration across toxicological groups for each flight-related trait structure or portion, we performed canonical variant analyses (CVA) and calculated Procrustes and Mahalanobis distances between toxicological groups, and evaluated the significance of these distances via a non-parametric test on the basis of permutations (1000 runs). These steps of morphometric analysis were performed using MorphoJ version 1.07a software [44]. We explored CS differentiation across toxicological groups after testing for normality of the CS data with the Shapiro–Wilks and one-way analysis of variance (ANOVA). Shapiro-Wilks and ANOVA analyses were done with the *R Stats Package*. For the study of variation for each flight-related trait measured, we performed analyses of morphological disparity. Disparity was calculated for size and shape measurements with the R package geomorph using the *morphol.disparity* function. Allometric relationships between forewing, membranous and stiff portion, and head shape and CS for females and males for each toxicological group were checked with a multivariate regression of Procrustes coordinates on CS. Regression results confirmed significant allometric effects only in 1 of the 24 multivariate regressions performed (*P* < 0.01), with relatively small allometric effect (7.11%).

To examine the covariation across forewing, stiff and membranous portions of the forewing, and heads across females and males of each toxicological group, we ran a series of.

pairwise comparison using partial least squares (PLS) between pairs of sets of landmarks (shape covariation) and CS (size covariation). We then statistically compared the effect sizes of PLS analyses. These steps of analysis were conducted using the *two.b.pls* and *compare.pls functions* of the package geomorph.

## Results

### Flight-related trait differentiation and variation across deltamethrin-resistant and susceptible *Triatoma infestans*

Dimorphisms in flight muscle were exhibited in the three toxicological groups (Table 2). The frequency of insects with and without muscle varied across toxicological groups only for females, and none exhibited significant differences between sexes. Females of the MR group exhibited the highest proportion of insects with flight muscle (86.21%) (Table 2).

**Table 2:** Number, percentage and results of the Chi-square goodness-of-fit test for females and males of *Triatoma infestans* with and without flight muscles according to the three toxicological studied groups

Toxicological group	Sex	Number (%) of individuals with flight muscles	Chi-square between sexes	Chi-square across toxicologic groups
HR	Female	12 (43.86%)	Chi-square: 0, p = 1	Chi-square: 25.51, p = 0.000
	Male	12 (43.86%)		Chi-square: 1.95, p = 0.3763
MR	Female	25 (86.21%)	Chi-square: 5.04, p = 0.3763	
	Male	29 (59.18%)		
S	Female	15 (28.30%)	Chi-square: 3.549, p = 0.06	
	Male	39 (54.93%)		

Toxicological groups names are as Table 1

For females, size of the wings, membranous and stiff portions, and head did not show significant differences across toxicological groups (*P* > 0.01 in all cases). For males, size of wings and membranous portion did not exhibit significant differences (*P* > 0.01 in both cases). However, size of the stiff portion and heads exhibited significant differences across toxicological groups (*P* < 0.01 in both cases). Post hoc Tukey tests showed that the susceptible phenotype exhibited stiff portions bigger than the other groups (S versus MR *P* < 0.01; S versus HR *P* < 0.05), while the susceptible and moderate resistant groups exhibited bigger heads than highly resistant group (S versus HR *P* < 0.01; MR versus HR *P* < 0.01).

**Fig. 2:**
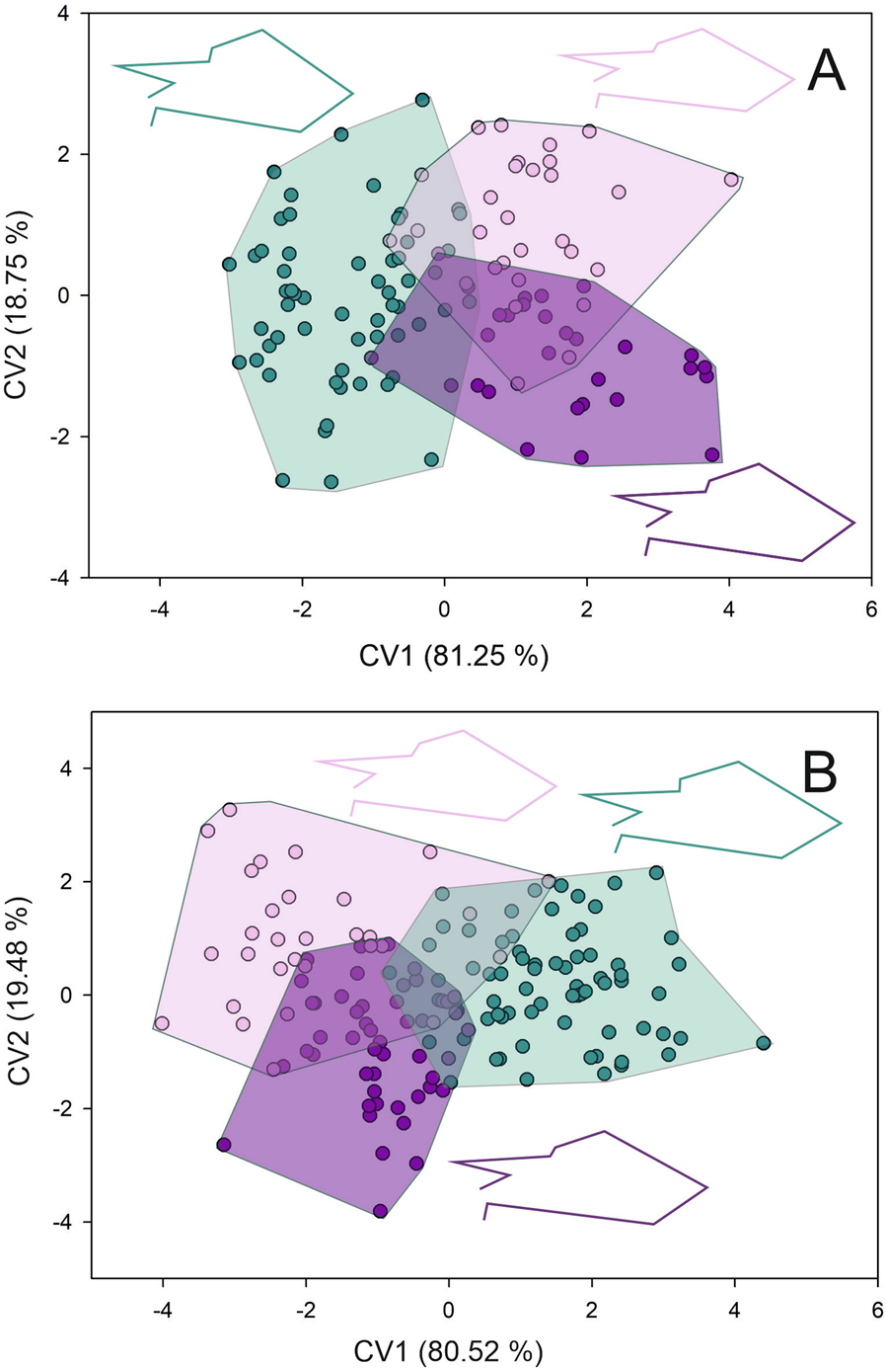
Factorial maps in the plane of the two first axes of a canonical variate analysis for shape measurements of the forewings from females (**A**) and males (**B**) of *T. infestans* belonging to the three toxicological phenotypes (susceptible (green dots), moderate resistant (violet dots), and highly resistant (lilacs dots)). Mean shape configuration for each group was shown

**Fig. 3:**
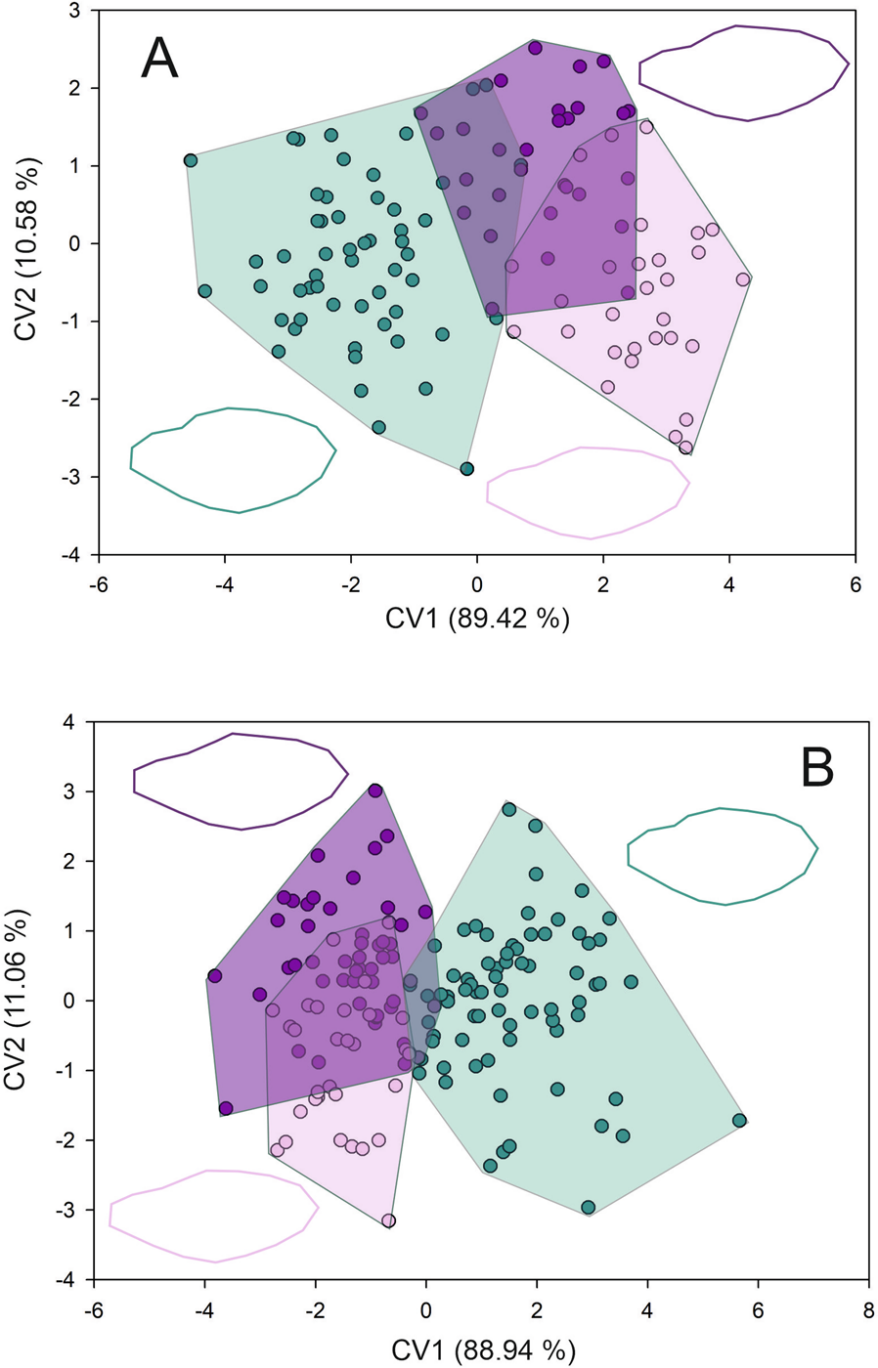
Factorial maps in the plane of the two first axes of a canonical variate analysis for shape measurements of the membranous portion of the forewing from females (**A**) and males (**B**) of *T. infestans* belonging to the three toxicological phenotypes (susceptible (green dots), moderate resistant (violet dots), and highly resistant (lilacs dots)). Mean shape configuration for each group was shown

**Fig. 4:**
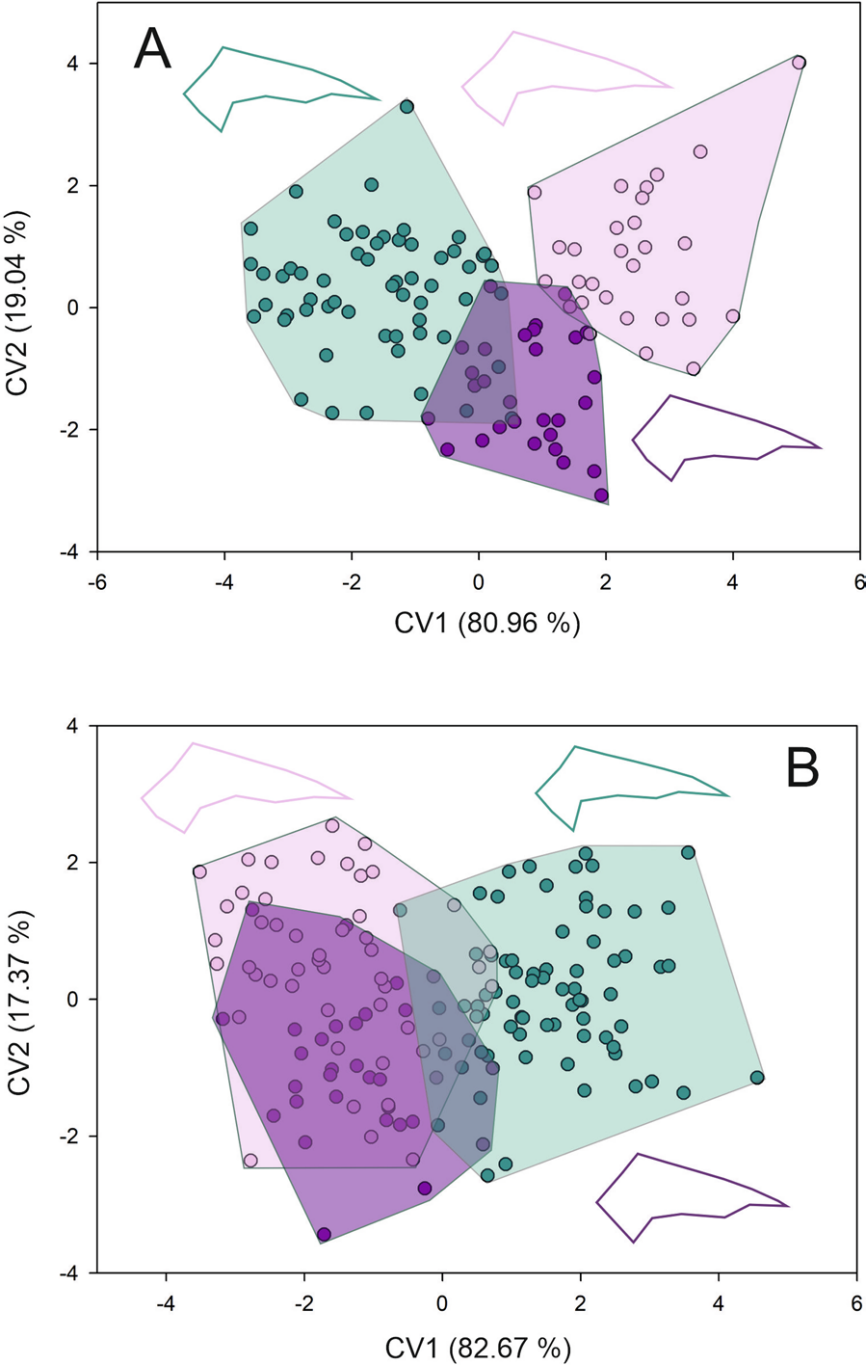
Factorial maps in the plane of the two first axes of a canonical variate analysis for shape measurements of the stiff portion of the forewing from females (**A**) and males (**B**) of *T. infestans* belonging to the three toxicological phenotypes (susceptible (green dots), moderate resistant (violet dots), and highly resistant (lilacs dots)). Mean shape configuration for each group was shown

**Fig. 5:**
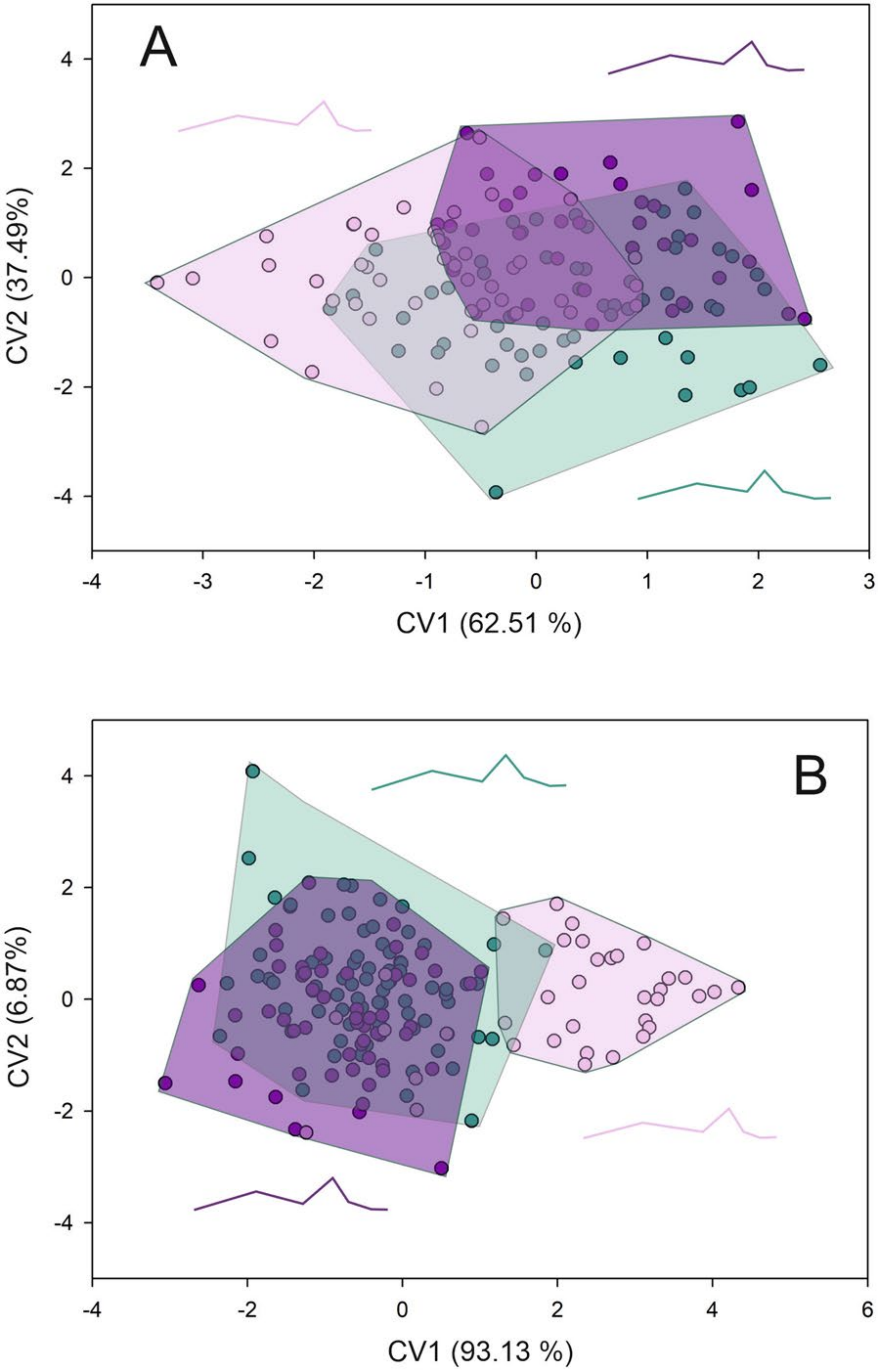
Factorial maps in the plane of the two first axes of a canonical variate analysis for shape measurements of the head from females (**A**) and males (**B**) of *T. infestans* belonging to the three toxicological phenotypes (susceptible (green dots), moderate resistant (violet dots), and highly resistant (lilacs dots)). Mean shape configuration for each group was shown

Forewing shape differed across toxicological groups, both for females and males. For females, the first two axes of the CVA accumulate 100% of the total differentiation (first axis 81.25%, second axis 18.75%) (Goodall’s *F*: 3.492, *P* = 0.0007). Both Procrustes and Mahalanobis distances exhibited significant differences across groups (Additional file 2 and 3) (Fig. 2a). The wings of susceptible females appear to be wider than those from females of resistant groups. Females from the highly resistant group exhibited narrower wings (Fig. 2a). For males, the first two axes of the CVA also accumulate 100% of the total differentiation (first axis 80.52%, second axis 19.48%) (Goodall’s *F*: 7.228, *P* < 0.0001). Males from the susceptible groups exhibited wider and longer wings than the resistant groups (Fig. 2b). Procrustes and Mahalanobis distances exhibited significant differences across toxicological groups (Additional file 2 and 3) (Fig. 2b). For membranous and stiff portions, the same tendency as for forewing shape was observed. For females, the first two axes of the CVA for the membranous portion accumulated 100% of the differentiation (89.42% and 10.58% for the first and second axes, respectively) (Goodall’s *F*: 14.951, *P* < 0.0001). Both Procrustes and Mahalanobis distances exhibited significant differences across toxicological groups (Additional file 2 and 3) (Fig. 3a). Females from the susceptible group exhibited a wider and shorter contour of the membranous portion than the other two groups (Fig. 3a). The CVA performed for males showed a Goodall’s *F* of 11.222 with a *P* < 0.0001. The first axis accumulated 88.94% of the differentiation while the second axis the 11.06%. Procrustes and Mahalanobis distances exhibited significant differences only between moderate resistant and highly resistant groups with susceptible group (Additional file 2 and 3) (Fig. 3b). For susceptible males, the contour of the membranous portion is wider and shorter than for the other two groups (Fig. 3b). For the stiff portion, the first two axes of the CVA performed for females showed that the first two axes accumulated 100% of the differentiation (80.96% and 19.04% for the first and second axes, respectively) (Goodall’s *F* of 4.731 with a *P* < 0.0001). Procrustes and Mahalanobis distances exhibited significant differences across toxicological groups (Additional file 2 and 3) (Fig. 4a). The contour of the stiff portion of the females from the resistant groups are wider than the susceptible females (Fig, 4a). For males, the first axis accumulated 82.67% and the second 17.37% (Goodall’s *F*: 11.188, *P* < 0.0001). Procrustes and Mahalanobis distances exhibited significant differences across toxicological groups, except for the Procrustes distances between moderate and resistant groups (Additional file 2 and 3). Males from the resistant groups exhibited a wider stiff portion than the susceptible males (Fig. 4b). Head shape exhibited the same tendency as forewing both for females and males (Goodall’s *F*: 7.926, *P* < 0.0001; Goodall’s *F*: 14.301, *P* < 0.0001, for females and males, respectively). For females, the first axis explained 62.51% of the variation and the second axis 37.49%, and Procrustes and Mahalanobis distances exhibited significant differences across toxicological groups (Additional file 2 and 3) (Fig. 5a). Head shape from the susceptible females showed a larger eye–ocelli distance and a shorter anteocular distance than the females of the other two groups (Fig. 5a). For males, the first two axes explained the total differentiation (93.13% and 6.87% for the first and second axes, respectively). Procrustes and Mahalanobis distances exhibited significant differences across toxicological groups (Additional file 2 and 3). As for males, susceptible males exhibited a greater eye–ocelli distance and a shorter anteocular distance than males of the other groups (Fig. 5b).

Morphological disparity across phenotypic toxicological groups for the size of the forewing, head, and membranous and stiff portions of the forewing did not show significant differences (Table 3). For shape measurements, morphological disparity analysis showed that only males exhibit disparity across toxicological groups (Table 4). Susceptible males showed significant disparity with either one or both resistant groups for the different measures; in all significant disparities, males from the susceptible group exhibited lower variance than resistant groups (results not shown).

**Table 3:** Pairwise absolute differences between variances (× 10^3^) derived from a morphological disparity analysis across phenotypic toxicological groups of *Triatoma infestans* for the size of the flight-related traits: forewing, head and membranous and stiff portions of the forewing.

Measurement	Sex	Toxicological group		
			MR	HR
Forewing	Female	S	0.195	0.297
		MR		0.492
	Male	S	0.436	0.783
		MR		0.348
Head	Female	S	0.051	0.067
		MR		0.118
	Male	S	0.310	0.162
		MR		0.471
Membranous portion	Female	S	1.104	1.929
		MR		3.033
	Male	S	1.603	2.141
		MR		0.538
Stiff portion	Female	S	0.639	0.150
		MR		0.789
	Male	S	0.774	1.273
		MR		0.499

Toxicological groups names are as Table 1

**Table 4:** Pairwise absolute differences between variances (× 10^3^) derived from a morphological disparity analysis across phenotypic toxicological groups of *Triatoma infestans* for the shape of the flight-related traits: forewing, head and membranous and stiff portions of the forewing.

Measurement	Sex	Toxicological group		
			MR	HR
Forewing	Female	S	0.226	0.066
		MR		0.160
	Male	S	0.379*	0.159
		MR		0.539^**^
Head	Female	S	0.226	0.066
		MR		0.160
	Male	S	0.071	0.482^**^
		MR		0.489^*^
Membranous portion	Female	S	1.556	0.550
		MR		1.006
	Male	S	2.562^*^	3.203^*^
		MR		0.064
Stiff portion	Female	S	0.424	0.518
		MR		0.942
	Male	S	1.965	7.834^*^
		MR		5.868

Toxicological groups names are as Table 1

^**^p < 0.01, * p < 0.05

### Covariation across flight-related traits in deltamethrin-resistant and susceptible *Triatoma infestans*

PLS analyses across the size of flight-related traits within each toxicological group are presented in Table 5. Almost all correlations across traits for the three toxicological groups showed significant association (except for the correlation between stiff portion of the forewings and heads for males of the highly resistant group) (Table 5). When comparing the magnitude of the PLS analyses through the effect size, between toxicological groups for females and males, none of the comparisons showed significantly different effect sizes (*P* > 0.01 in all cases, results not shown). For shape, PLS analyses across flight-related traits for females showed that the highly resistant group showed only two significant PLS, between head and membranous portion of the wing and between membranous and stiff portions (Table 6), while for the other two toxicological groups, all the PLS analyses showed significant associations (Table 6). Males of the susceptible group did not show significant association between head and forewing and membranous portion of the forewing and between membranous and stiff portion (Table 6). For moderate resistant males, heads were not significantly correlated with forewing or its parts, and for highly resistant males, no significant correlation was present except for forewing and head (Table 6). When comparing the magnitude of the PLS, for females the PLS analysis between the forewing and membranous portion between the susceptible and highly resistant groups showed significant differences (*P* < 0.01). Moreover, the PLS analysis between the forewing and stiff portion showed significant differences between the susceptible and highly resistant groups and between moderate and highly resistant groups (*P* < 0.01 in both analyses). For males, the PLS analysis between the forewing and membranous portion showed significant differences between the susceptible and highly resistant groups (*P* < 0.01). The PLS analysis between the forewing and stiff portion showed significant differences between the susceptible and highly resistant group and between moderate and highly resistant groups (*P* < 0.0001 and *P* < 0.01, respectively). Head and stiff portion showed significant differences between the moderate and highly resistant groups (*P* < 0.05).

**Table 5:** Results of the partial least square analyses (PLS) between size measurement of forewing, head and membranous and stiffy portions across toxicological groups for females and males of *Triatoma infestans*

Toxicological group	Sex	Measurement	Head	Membranous portion	Stiff portion
			r-PLS	r-PLS	r-PLS
S	Female	Forewing	0.649^***^	0.917^***^	0.803^***^
		Head		0.614^***^	0.662^***^
		Membranous portion			0.832^***^
	Male	Forewing	0.585^***^	0.931^***^	0.893^***^
		Head		0.572^***^	0.600^***^
		Membranous portion			0.867^***^
MR	Female	Forewing	0.565^**^	0.899^***^	0.889^***^
		Head		0.589^**^	0.449*
		Membranous portion			0.821^***^
	Male	Forewing	0.655***	0.939^***^	0.877^***^
		Head		0.572^***^	0.667^***^
		Membranous portion			0.833^***^
HR	Female	Forewing	0.558**	0.919^***^	0.837^***^
		Head		0.479^**^	0.502^**^
		Membranous portion			0.878^***^
	Male	Forewing	0.585**	0.926^***^	0.682^***^
		Head		0.537^**^	0.340
		Membranous portion			0.765^***^

Toxicological groups names are as Table 1

^***^p < 0.001, ** p < 0.01, * p < 0.05

**Table 6:** Results of the partial least square analyses (PLS) between shape measurement of forewing, head and membranous and stiffy portions across toxicological groups for females and males of *T. infestans*

Toxicological group	Sex	Measurement	Head	Membranous portion	Stiff portion
			r-PLS	r-PLS	r-PLS
S	Female	Forewing	0.531^*^	0.742^***^	0.819^***^
		Head		0.614^***^	0.374
		Membranous portion			0.339
	Male	Forewing	0.420	0.699^***^	0.865^***^
		Head		0.386	0.567^***^
		Membranous portion			0.427^*^
MR	Female	Forewing	0.699^**^	0.643^*^	0.944^***^
		Head		0.691^**^	0.676^**^
		Membranous portion			0.652^**^
	Male	Forewing	0.436	0.700^***^	0.864^***^
		Head		0.455	0.489
		Membranous portion			0.520^*^
HR	Female	Forewing	0.540	0.510	0.584^*^
		Head		0.654^*^	0.545
		Membranous portion			0.653
	Male	Forewing	0.689^**^	0.606	0.506
		Head		0.433	0.595
		Membranous portion			0.597

Toxicological groups names are as Table 1

^***^p < 0.0001, ** p < 0.001, * p < 0.05

## Discussion

Flight is a key active dispersal mechanism in triatomines, playing a significant role in the spread and survival of species such as *T. infestans* [30, 31]. The evolution of dispersal traits in a population is driven by changes in locomotor and navigation abilities [45]. In *T. infestans*, flight dispersal is influenced by a combination of factors, including wing morphology, head structure, and the development of flight muscle. These morphological traits not only contribute to the insect’s ability to disperse through flight but also interact with insecticide resistance. For instance, previous studies (e.g., [29]) have suggested that certain morphological adaptations may facilitate or hinder dispersal in populations with varying levels of insecticide resistance, with potential changes in the dynamics of vector spread. The size and shape of wings are crucial for flight performance. In hemipterans, the forewings, or hemelytra, consist of a stiff proximal region and a more flexible membranous apex. This morphological design is integral to their function: the flexible portion allows for deformation under aerodynamic forces, while the stiff portion provides structural support and restricts unwanted deformation [46]. Therefore, the shape, size, and development of the forewing parts are critical determinants of flight efficiency. In our study, we found that male *T. infestans* from susceptible (S) populations exhibited a larger stiff portion of the forewing compared with moderately resistant (MR) and highly resistant (HR) groups. Shape variation analysis revealed that S females had wider forewings than resistant females, and HR females exhibited more narrowly shaped wings with a wider contour of the stiff portion. In males, susceptible individuals had wider and longer wings compared with resistant groups, which also displayed a broader stiff portion. These differences in wing morphology support the hypothesis that longer, narrower wings are more efficient for long-distance flights due to their increased capacity for deformation with lower energy costs [46]. Previous studies have similarly shown that pyrethroid-resistant *T. infestans* exhibit smaller wings and altered wing shapes—narrower and longer wings compared with susceptible individuals—which can be attributed to the tradeoffs associated with resistance [29].

In *T. garciabesi*, studies have shown that wing shape variations between different genetic lineages are associated with environmental factors and dispersal patterns, with narrower and longer wings in the Western lineage and broader, shorter wings in the Eastern lineage [47]. These variations likely reflect different flight capacities tied to the specific ecological contexts of each lineage. Similarly, in *T. infestans*, the differences observed in wing morphology between susceptible and resistant groups suggest distinct dispersal abilities. These morphological changes may be pleiotropic effects of pyrethroid resistance, where the biochemical mechanisms conferring resistance interfere with the development of flight-related traits. The tradeoff between resistance and dispersal capacity is a critical evolutionary consideration, as resistance could compromise flight efficiency, and by extension, dispersal potential. The biochemical mechanisms behind insecticide resistance, such as the overproduction of detoxifying enzymes, may deplete energy reserves and impose metabolic costs [14, 48]. These costs can reduce the energy available for other physiological functions, such as flight muscle development and reproduction, leading to tradeoffs in traits critical for survival and reproduction [48]. Our findings support this idea, with resistant males showing smaller head sizes and altered forewing morphology, which may reflect a compromised flight capacity due to the energy allocation toward resistance mechanisms. These results highlight the potential adaptive costs of resistance, where energy invested in detoxification and resistance mechanisms detracts from the energy available for other key functions such as dispersal and reproduction.

Previous studies have shown that variations in head shape are linked to flight capacity in insects [49, 50]. In several species, changes in head morphology have been associated with flight performance (e.g., [45, 49]). In triatomines such as *Mepraia spinolai* and *T. guasayana*, specific head traits, including narrow heads and well-developed compound eyes, are thought to facilitate flight dispersal [49–51]. For *M. spinolai* and *T. guasayana*, macropterous individuals with more convex compound eyes and larger interocular distances may have enhanced navigational ability and orientation during flight compared with micropterous individuals [49, 50]. In *T. garciabesi*, changes in head size and shape were observed, particularly in males, with more convex eyes linked to higher levels of anthropogenic disturbance [52]. For *T. infestans*, resistant individuals from both sexes had smaller heads, with changes in shape such as reduced anteocular distance and smaller eyes in resistant strains (RR) [29]. In *T. infestans*, it is possible to use wing size as a proxy of overall organism size [40, 53]. Our results provide evidence that pyrethroid resistance in *T. infestans* is associated with smaller body size. Additionally, for *T. infestans*, allometric relationships between body size and physiological traits such as blood meal content and female fecundity have been documented [54]. In *T. infestans*, these changes in head size and shape may also reflect the pleiotropic effects of resistance, further suggesting that resistant individuals may face a tradeoff between dispersal capacity and resistance.

Our findings also indicate that flight muscle development, an essential determinant of flight capacity, did not show significant differences between male and female populations. However, both MR and HR females had a higher proportion of individuals with well-developed flight muscles than S ones, which suggests that resistance may not directly impede the development of flight muscles. Whether having developed muscles translated into more profound reproductive costs deserves further studies. It has been well documented that resistant populations often exhibit reduced dispersal ability and reproductive success compared with susceptible populations [19–21]. While resistant individuals may invest more energy in maintaining resistance mechanisms, this could reduce their capacity for reproductive output and dispersal. In contrast, susceptible populations may allocate more energy toward reproduction, as they do not incur the metabolic costs of resistance, suggesting a potential tradeoff between resistance and reproductive success or flight dispersal.

The study of intraspecific variation in populations is fundamental to understanding the evolutionary forces shaping the variability of traits such as dispersal capacity and resistance. Selection pressures on functional traits, such as wings and flight muscles, can lead to reduced phenotypic variance when traits are optimized for a particular function [55, 56]. Our results suggest that selection on dispersal-related traits, such as wing and head morphology, is relaxed or altered in resistant populations of *T. infestans*, resulting in greater morphological variation in these traits compared with susceptible populations. This variation may reflect a tradeoff between flight dispersal ability and the energy invested in resistance mechanisms. The balance between these competing selective pressures is crucial for understanding the long-term ecological and evolutionary dynamics of *T. infestans* populations, especially in the context of insecticide resistance management.

Our results showed a general reduction in covariation between flight-related traits for pyrethroid-resistant individuals, with the magnitude of these covariations decreasing in both males and females. These findings, along with previous data, support the hypothesis that pleiotropic effects of insecticide resistance influence the size, shape, variation, and covariation of flight-related traits, especially in males.

The relationship between flight-related traits and insecticide resistance is complex. While resistance confers immediate survival advantages in treated environments, the associated morphological and behavioral changes may impose costs that negatively impact overall fitness and dispersion. Our study suggests that resistant individuals may exhibit reduced flight capacity, which could translate in their natural habitat to differences in active dispersal capacity compared with susceptible individuals. These differences have implications for population dynamics, the evolution of insecticide resistance, and the effectiveness of vector control measures in disease epidemiology. As *T. infestans* populations adapt to insecticide resistance, understanding how these adaptations affect flight-related traits is crucial for predicting changes in dispersal patterns and ecological roles. The interaction between selective pressures on flight traits and the pleiotropic effects of resistance is central to understanding the evolutionary trajectories of insect populations. Consequently, natural selection may favor individuals that strike a balance between resistance and flight capacity. It remains challenging to determine whether the morphological differences observed represent an adaptive cost for resistant insects.

While our study focused primarily on the morphometric adaptations of flight-related traits and insecticide resistance, it is likely that different ecological contexts—such as variations in pesticide exposure, host availability, and different domestic and peridomestic ecotopes—play a significant role in shaping the evolutionary trajectories of dispersal characteristics in *T. infestans* populations (e.g., [37, 57–60]). Analyzing the relationship between resistance and dispersal across different habitats would offer valuable insights for Chagas disease control strategies. The characteristics of domestic environments, where low-dose insecticide application could be more frequent [61], could translate into a stronger selective pressure on resistance traits. In contrast, peridomestic environments, which often feature more heterogeneous conditions and irregular pesticide exposure, might impose different selective pressures that influence dispersal traits, such as flight capacity and hemelytra morphology. Speculatively, it is possible that these habitat-driven differences could lead to the development of distinct morphotypes within populations, potentially changing the relationship between resistance and dispersal. Therefore, integrating environmental variables into future research would provide a more comprehensive understanding of the adaptive processes involved in *T. infestans* resistance and dispersal, helping to avoid an oversimplified interpretation of these complex evolutionary dynamics. While our study provides valuable insights into the variation in flight-related traits associated with differences in insecticide resistance profiles, it is inherently observational and cannot identify the specific resistance mechanisms underlying the distinct toxicological groups, particularly the characteristics that define the MR group. Additionally, individuals from the HR group were sourced from Castelli municipality, while the MR and S groups came from Avia Terai municipality, approximately 80 km apart. Despite this geographical difference, our results suggest that the toxicological groups differ from one another, even when originating from the same locality, as observed between the S and MR groups. We also found common patterns in the flight-related trait variations between the MR and HR groups, which were distinct from those of the S group. While this limitation should be acknowledged, it does not diminish the fact that flight-related traits varied significantly across the toxicological groups, potentially influencing dispersal capacity in the resistant groups. Further experimental studies are needed to better understand the relationship between variations in flight-related traits and dispersal capacity in *T. infestans*.

## Conclusions

Our study reveals that pyrethroid resistance in *T. infestans* is linked to morphological changes in flight-related traits, including smaller head size and altered wing shape. These adaptations suggest a tradeoff between resistance and flight capacity, with energy allocated to resistance mechanisms potentially reducing the energy available for flight and other physiological functions. The reduction in covariation between flight-related traits in resistant individuals supports the idea of pleiotropic effects, where selection for resistance may disrupt the integration of traits critical for dispersal. While resistant individuals may have an advantage in treated environments, their compromised flight capacity could limit long-distance dispersal, potentially affecting population dynamics and vector control efforts. Our findings suggest that resistant insects might be more suited to short-distance dispersal, while susceptible individuals are better adapted for longer flights. These insights highlight the importance of considering the evolutionary tradeoffs in insecticide resistance when developing pest management strategies and understanding the long-term dynamics of *T. infestans* populations.

## Supplementary Information

Additional file 1. Raw coordinates of the landmark and semilandmark configuration for flight-related traits: forewing, membranous portion of the forewing, stiff portion of the forewing, and head

Additional file 2. Procrustes distances across phenotypic toxicological groups of *Triatoma infestans* for the shape of the flight-related traits: forewing, head, and membranous and stiff portions of the forewing. Toxicological group names are as in Table 1.

Additional file 3. Mahalanobis distances across phenotypic toxicological groups of *Triatoma infestans* for the shape of the flight-related traits: forewing, head and membranous and stiff portions of the forewing. Toxicological group names are as in Table 1.

## Data Availability

Data are provided within the manuscript or supplementary information files.

## References

[1] PAHO (Pan American Health Organization). 2014. Chagas disease. https://www.paho.org/en/topics/chagas-disease. Accessed on Nov 2024.

[2] WHO (World Health Organization). 2024. Chagas disease. https://www.who.int/health-topics/chagas-disease#:~:text=Chagas%20disease%2C%20also%20known%20as,cruzi. Accessed on Nov 2024.

[3] Mougabure-Cueto G, Picollo MI. Insecticide resistance in vector chagas disease: evolution, mechanisms and management. Acta Trop. 2015;149:70–85. 10.1016/j.actatropica.2015.05.014. 26003952 10.1016/j.actatropica.2015.05.014

[4] Kliot A, Ghanim M. Fitness costs associated with insecticide resistance. Pest Manag Sci. 2012;68:1431–7. 10.1002/ps.3395. 22945853 10.1002/ps.3395

[5] Rivero A, Vézilier J, Weill M, Read AF, Gandon S. Insecticide control of vector-borne diseases: when is insecticide resistance a problem? PLoS Pathog. 2010;6:e1001000. 10.1371/journal.ppat.1001000. 20700451 10.1371/journal.ppat.1001000 PMC2916878

[6] Picollo MI, Vassena C, Santo Orihuela P, Barrios S, Zaidemberg M, Zerba E. High resistance to pyrethroid insecticides associated with ineffective field treatments in *Triatoma infestans* (Hemiptera: Reduviidae) from northern Argentina. J Med Entomol. 2005;42:637–42. 10.1093/jmedent/42.4.637. 16119553 10.1093/jmedent/42.4.637

[7] Gurevitz JM, Gaspe MS, Enríquez GF, Vassena CV, Alvarado-Otegui JA, Provecho YM, Mougabure Cueto GA, Picollo MI, Kitron U, Gürtler RE. Unexpected failures to control Chagas disease vectors with pyrethroid spraying in northern Argentina. J Med Entomol. 2012;49:1379–86. 10.1603/me11157. 23270166 10.1603/me11157 PMC3760256

[8] Vassena C, Picollo MI, Santo Orihuela P, Zerba E. Desarrollo y manejo de la resistencia a insecticidas piretroides en *Triatoma infestans*: situación en Bolivia. In: Rojas Cortez M editor. Triatominos de Bolivia y la enfermedad de Chagas. La Paz, Bolivia: Ministerio de Salud y Deportes, Unidad de Epidemiología, Programa Nacional de Chagas*.* 2007; 229–258.

[9] Fronza G, Toloza AC, Picollo MI, Spillmann C, Mougabure-Cueto GA. Geographical variation of deltamethrin susceptibility of *Triatoma infestans* (Hemiptera: Reduviidae) in Argentina with emphasis on a resistant focus in the Gran Chaco. J Med Entomol. 2016;53:880–7. 10.1093/jme/tjw056. 27113106 10.1093/jme/tjw056

[10] Gaspe MS, Cardinal MV, Fernández MP, Vassena CV, Santo-Orihuela PL, Enriquez GF, et al. Improved vector control of *Triatoma infestans* limited by emerging pyrethroid resistance across an urban-to-rural gradient in the Argentine Chaco. Parasit Vectors. 2021;14:437. 10.1186/s13071-021-04942-9. 34454569 10.1186/s13071-021-04942-9 PMC8401064

[11] Fronza G, Toloza AC, Picollo MI, Carbajo AE, Rodríguez S, Mougabure- Cueto GA. Modelling the association between deltamethrin resistance in *Triatoma infestans* populations of the Argentinian Gran Chaco region with environmental factors. Acta Trop. 2019;194:53–61. 10.1016/j.actatropica.2019.03.021. 30898614 10.1016/j.actatropica.2019.03.021

[12] McKenzie JA. Ecological and evolutionary aspects of insecticide resistance. California: Academic Press, Inc.; 1996.

[13] Onstad DW, Guse CA. Concepts and complexities of population genetics. In: Onstad DW, editor. Insect resistance management: biology, economics and predictions. USA: Elsevier; 2008. p. 69–88.

[14] Roush RT, McKenzie JA. Ecological genetics of insecticide and acaricide resistance. Annu Rev Entomol. 1987;32:361–80. 10.1146/annurev.en.32.010187.002045. 3545056 10.1146/annurev.en.32.010187.002045

[15] Kliot A, Ghanim M. Fitness costs associated with insecticide resistance. Pest Manag Sci. 2012;68:1431–7. 10.1002/ps.3395. 22945853 10.1002/ps.3395

[16] Maza VA, Nattero J, Gaspe MS, Cardinal MV. Extended stage duration and diminished fecundity in deltamethrin-resistant *Triatoma infestans* (Klug, 1834) of the Argentinean Chaco. Med Vet Entomol. 2023;37:834–44. 10.1111/mve.12689. 37658694 10.1111/mve.12689

[17] Germano MD, Picollo MI. Reproductive and developmental costs of deltamethrin resistance in the Chagas disease vector *Triatoma infestans*. J Vector Ecol. 2015;40:1–7. 10.1111/jvec.12132. 10.1111/jvec.12132 26047184

[18] Germano MD, Picollo MI. Demographic effects of deltamethrin resistance in the Chagas disease vector *Triatoma infestans*. Med Vet Entomol. 2016;30:416–25. 10.1111/mve.12196. 27677531 10.1111/mve.12196

[19] Lobbia PA, Rodríguez C, Mougabure-Cueto G. Effect of nutritional state and dispersal on the reproductive efficiency in *Triatoma infestans* (Klug, 1834) (Hemiptera: reduviidae: triatominae) susceptible and resistant to deltamethrin. Acta Trop. 2019;191:228–38. 10.1016/j.actatropica.2019.01.012. 30653943 10.1016/j.actatropica.2019.01.012

[20] Lobbia PA, Rodríguez C, Mougabure-Cueto G. Effect of reproductive state on active dispersal in *Triatoma infestans* (Klug, 1834) (Hemiptera: Reduviidae: Triatominae) susceptible and resistant to deltamethrin. Acta Trop. 2019;196:7–14. 10.1016/j.actatropica.2019.05.002. 31054918 10.1016/j.actatropica.2019.05.002

[21] Lobbia PA, Mougabure-Cueto G. Active dispersal in *Triatoma infestans* (Klug, 1834) (Hemiptera: Reduviidae: Triatominae): effects of nutritional status, the presence of a food source and the toxicological phenotype. Acta Trop. 2020;204:105345. 10.1016/j.actatropica.2020.105345. 31954136 10.1016/j.actatropica.2020.105345

[22] Lobbia P, Calcagno J, Mougabure-Cueto G. Excretion/defecation patterns in *Triatoma infestans* populations that are, respectively, susceptible and resistant to deltamethrin. Med Vet Entomol. 2018;32:311–22. 10.1111/mve.12298. 29430671 10.1111/mve.12298

[23] May-Concha I, Remón C, Mougabure-Cueto G. Behavioral response mediated by feces in *Triatoma infestans* (Hemiptera: Reduviidae: Triatominae) susceptible and resistant to deltamethrin. Acta Trop. 2020;206:105442. 10.1016/j.actatropica.2020.105442. 32171756 10.1016/j.actatropica.2020.105442

[24] Guanuco AP, Davies C, Poma HR, Gentile AG, Cardozo RM. Pyrethroid resistant and susceptible *Triatoma infestans* (Klug, 1834) (Hemiptera, Triatominae): analysis of their vectorial characteristics by metacyclogenesis, feeding/defecation patterns, and parasite load. Parasitologia. 2022;2:255–65. 10.3390/parasitologia2040022.

[25] Mougabure-Cueto G, Lobbia PA. Estado de la resistencia a insecticidas en *Triatoma infestans* de Argentina. Rev Salud Ambient. 2021;21:137–46.

[26] Willmore K, Young N, Richtsmeier J. Phenotypic variability: its components, measurement and underlying developmental processes. Evol Biol. 2007;34:99–120. 10.1007/s11692-007-9008-1.

[27] Klingenberg C. Studying morphological integration and modularity at multiple levels: concepts and analysis. Philos Trans R Soc Lond B. 2014;369:20130249. 10.1098/rstb.2013.0249. 25002695 10.1098/rstb.2013.0249 PMC4084535

[28] Nattero J, Mougabure-Cueto G, Gürtler RE. Sublethal effects of a pyrethroid insecticide on cuticle thickness, wing size, and shape in the main vector *Triatoma infestans*. Med Vet Entomol. 2022;36:397–407. 10.1111/mve.12600. 35946595 10.1111/mve.12600

[29] Hernández ML, Dujardin JP, Villacís AG, Yumiseva CA, Remón C, Mougabure-Cueto G. Resistance to deltamethrin in *Triatoma infestans* (Hemiptera: Reduviidae): does it influence the phenotype of antennae, wings, and heads? Acta Trop. 2023;245:106976. 10.1016/j.actatropica.2023.106976. 37352997 10.1016/j.actatropica.2023.106976

[30] Wisnivesky-Colli C, Gürtler RE, Solarz ND, Schweigmann NJ, Pietrokovsky SM, Alberti A, et al. Dispersive flight and house invasion by *Triatoma guasayana* and *Triatoma sordida* in Argentina. Mem Inst Oswaldo Cruz. 1993;88:27–32. 8246755 10.1590/s0074-02761993000100006

[31] Galvão C, Da Silva RD, Jurberg J, Carcavallo R. Início da atividade de vôo en Triatoma infestans (Klug 1834) e *T. melanosoma* Martínez Olmedo and Carcavallo 1987 (Hemiptera Reduviidae). Mem Inst Oswaldo Cruz. 2001;96:137–40. 10.1590/S0074-02762001000100017. 11285486 10.1590/s0074-02762001000100017

[32] Gurevitz JM, Ceballos LA, Kitron U, Gürtler RE. Flight initiation of *Triatoma infestans* (Hemiptera: Reduviidae) under natural climatic conditions. J Med Entomol. 2006. 10.1603/0022-2585(2006)043[0143:fiotih]2.0.co;2. 16619592 10.1603/0022-2585(2006)043[0143:fiotih]2.0.co;2 PMC1894897

[33] Schofield CJ, Lehane MJ, McEwen P, Catala SS, Gorla DE. Dispersive flight by *Triatoma infestans* under natural climatic conditions in Argentina. Med Vet Entomol. 1992;6:51–6. 10.1111/j.1365-2915.1992.tb00035.x. 1600228 10.1111/j.1365-2915.1992.tb00035.x

[34] Schweigmann N, Vallve S, Muscio O, Ghillini N, Alberti A, Wisnivesky-Colli C. Dispersal flight by *Triatoma infestans* in an arid area of Argentina. Med Vet Entomol. 1988;2:401–4. 10.1111/j.1365-2915.1988.tb00215.x. 2980200 10.1111/j.1365-2915.1988.tb00215.x

[35] Gaspe MS, Fernández MP, Cardinal MV, Enriquez GF, Rodríguez-Planes LI, Macchiaverna NP, et al. Urbanisation, risk stratification and house infestation with a major vector of Chagas disease in an endemic municipality of the Argentine Chaco. Parasit Vectors. 2020;13:316. 10.1186/s13071-020-04182-3. 32552813 10.1186/s13071-020-04182-3 PMC7302373

[36] World Health Organization (WHO). Workshop on the insecticide effect evaluation in Triatominos. Acta Toxicol Argent. 1994;2:29–58.

[37] Schachter-Broide J, Dujardin J-P, Kitron U, Gürtler RE. Spatial structuring of *Triatoma infestans* (Hemiptera, Reduviidae) populations from northwestern Argentina using wing geometric morphometry. J Med Entomol. 2004;41:643–9. 10.1603/0022-2585-41.4.643. 15311455 10.1603/0022-2585-41.4.643 PMC1351235

[38] Gunz P, Mitteroecker P. Semilandmarks: a method for quantifying curves and surfaces. Hystrix. 2013;24:103–9. 10.4404/hystrix-24.1-6292.

[39] Rohlf FJ. TpsDig, digitize landmarks and outlines version 2.31. New York: Department of ecology and evolution State University of New York at Stony Brook. 2017.

[40] Gaspe MS, Schachter-Brodie J, Gurevitz JM, Kitron U, Gürtler RE, Dujardin J-P. Microgeographic spatial structuring of *Triatoma infestans* (Hemiptera: Reduviidae) populations using wing geometric morphometry in the argentine Chaco. J Med Entomol. 2012;49:504–14. 10.1603/ME11176. 22679857 10.1603/me11176 PMC3566984

[41] Nattero J, Carbajal de la Fuente AL, Piccinali RV, Cardozo M, Rodríguez CS, Crocco LB. Characterization of melanic and non-melanic forms in domestic and peridomestic populations of Triatoma infestans (Hemiptera: Reduviidae). Parasit Vectors. 2020;13:47. 10.1186/s13071-020-3912-y. 32014037 10.1186/s13071-020-3912-y PMC6998255

[42] Dryden IL, Mardia KV. Statistical shape analysis. Chichester: Wiley; 1998.

[43] Nattero J, Piccinali RV, Fiad FG, Cano F, Carbajal de la Fuente AL. Relationship between flight muscle dimorphism and wing morphometry in *Triatoma infestans* (Klug, 1834) (Hemiptera, Reduviidae, Triatominae). Front Ecol Evol. 2023;11:1211219. 10.3389/fevo.2023.1211219.

[44] Klingenberg CP. MorphoJ: an integrated software package for geometric morphometrics. Mol Ecol Resour. 2011;11:353–7. 10.1111/j.1755-0998.2010.02924.x. 21429143 10.1111/j.1755-0998.2010.02924.x

[45] Turlure C, Schtickzelle N, Van Dyck H, Seymoure B, Rutowski R. Flight morphology, compound eye structure and dispersal in the bog and the cranberry fritillary butterflies: an inter- and intraspecific comparison. PLoS ONE. 2016;11:e0158073. 10.1371/journal.pone.0158073. 27336590 10.1371/journal.pone.0158073 PMC4919012

[46] Wootton RJ. Functional wing morphology in Hemiptera systematics. In: Schaefer CW, editor. Studies in Hemipteran phylogeny Entomological Society of America. Washington: . SPIE; 1996. p. 179–98.

[47] Verly T, Pita S, Carbajal de la Fuente AL, Burgueño-Rodríguez G, Piccinali RV, Fiad FG, et al. Relationship between genetic diversity and morpho-functional characteristics of flight-related traits in Triatoma garciabesi (Hemiptera: Reduviidae). Parasit Vectors. 2024;17:145. 10.1186/s13071-024-06211-x. 38500121 10.1186/s13071-024-06211-x PMC10949591

[48] Rivero A, Magaud A, Nicot A, V´ezilie J. Energetic cost of insecticide resistance in *Culex pipiens* mosquitoes. J Med Entomol. 2011;48:694–700. 10.1603/me10121. 21661333 10.1603/me10121

[49] Hernández ML, Dujardin JP, Gorla DE, Catalá SS. Can body traits, other than wings, reflect the flight ability of Triatominae bugs? Rev Soc Bras Med Trop. 2015;48:682–91. 10.1590/0037-8682-0249-2015. 26676492 10.1590/0037-8682-0249-2015

[50] Hernández ML, Espinoza J, Gomez M, Gorla D. Morphological changes associated with brachypterous *Triatoma guasayana* (Hemiptera, Reduviidae) and their relationship with flight. Int J Trop Insect Sci. 2020;40:413–21. 10.1007/s42690-019-00092-9.

[51] Gigena GV, Rodríguez CS, Fiad FG, Hernández ML, Carbajal-de-la-Fuente AL, Piccinali RV, et al. Phenotypic variability in traits related to flight dispersal in the wing dimorphic species *Triatoma guasayana*. Parasit Vectors. 2023;16:8. 10.1186/s13071-022-05570-7. 36624528 10.1186/s13071-022-05570-7 PMC9830765

[52] Fiad FG, Cardozo M, Nattero J, Gigena GV, Gorla DE, Rodríguez CS. Association between environmental gradient of anthropization and phenotypic plasticity in two species of triatomines. Parasit Vectors. 2024;17:169. 10.1186/s13071-024-06258-w. 38566228 10.1186/s13071-024-06258-w PMC10986143

[53] Dujardin J-P, Slice DE. Contributions of morphometrics to medical entomology. In: Tibayrenc M, editor. Encyclopedia of Infectious Diseases. New Jersey: John Wiley & Sons Inc; 2006. p. 433–46.

[54] Gürtler RE, Fernández MP, Cecere MC, Cohen JE. Body size and hosts of *Triatoma infestans* populations affect the size of bloodmeal contents and female fecundity in rural northwestern Argentina. PLoS Negl Trop Dis. 2017;11:e0006097. 10.1371/journal.pntd.0006097. 29211791 10.1371/journal.pntd.0006097 PMC5734792

[55] Travis JMJ, Dytham C. Dispersal evolution during invasion. Evol Ecol Res. 2002;4:1119–29.

[56] Holt RD. On the evolutionary ecology of species’ ranges. Evol Ecol Res. 2003;5:159–78.

[57] Nattero J, Leonhard G, Gürtler RE, Crocco LB. Evidence of selection on phenotypic plasticity and cost of plasticity in response to host-feeding sources in the major Chagas disease vector *Triatoma infestans*. Acta Trop. 2015;152:237–44. 10.1016/j.actatropica.2015.09.022. 26433077 10.1016/j.actatropica.2015.09.022

[58] Nattero J, Dujardin J-P, Fernández MP, Gürtler RE. Host-feeding sources and habitats jointly affect wing developmental stability depending on sex in the major Chagas disease vector *Triatoma infestans*. Infect Genet Evol. 2015;36:539–46. 10.1016/j.meegid.2015.08.032. 26318543 10.1016/j.meegid.2015.08.032

[59] Nattero J, Piccinali RV, Gaspe MS, Gürtler RE. Fluctuating asymmetry and exposure to pyrethroid insecticides in *Triatoma infestans* populations in northeastern Argentina. Infect Gen Evol. 2019;74:103925. 10.1016/j.meegid.2019.103925. 10.1016/j.meegid.2019.103925 31220610

[60] Nattero J, Mougabure-Cueto G, Debat V, Gürtler RE. Phenotypic plasticity, canalisation and developmental stability of *Triatoma infestans* wings: effects of a sublethal application of a pyrethroid insecticide. Parasit Vectors. 2021;14:355. 10.1186/s13071-021-04857-5. 34229739 10.1186/s13071-021-04857-5 PMC8259426

[61] Cecere MC, Gaspe MS, Macchiaverna NP, Enriquez GF, Alvedro A, Laiño MA, et al. Slow recovery rates and spatial aggregation of *Triatoma infestans* populations in an area with high pyrethroid resistance in the Argentine Chaco. Parasit Vectors. 2024;17:287. 10.1186/s13071-024-06366-7. 38956689 10.1186/s13071-024-06366-7 PMC11220979

