# Supplementary material for: Morphofunctional characteristics of flight-related traits in deltamethrin-resistant and susceptible Triatoma infestans (Klug, 1834) of the Argentinean Chaco
Source: Parasit Vectors. 2025 Mar 6;18:92. doi: 10.1186/s13071-025-06678-2 (PMC11883966; doi:10.1186/s13071-025-06678-2)
Supplement: Supplementary file 2 — Additional file 2. Procrustes distances across phenotypic toxicological groups of Triatoma infestans for the shape of the flight-related traits: forewing, head, and membranous and stiff portions of the forewing. Toxicological group names are as in Table 1. [file 13071_2025_6678_MOESM2_ESM.docx]

Additional file 2

| **Measurement** | **Sex** | **Toxicological group**  **MR HR** | | |
| --- | --- | --- | --- | --- |
| Forewing | Female | S | 0.017* | 0.014* |
|  |  | MR |  | 0.016* |
|  | Male | S | 0.017*** | 0.022*** |
|  |  | MR |  | 0.015** |
| Head | Female | S | 0.104* | 0.182*** |
|  |  | MR |  | 0.020*** |
|  | Male | S | 0.010* | 0.024*** |
|  |  | MR |  | 0.029*** |
| Membranous portion | Female | S | 0.020*** | 0.027*** |
|  |  | MR |  | 0.010** |
|  | Male | S | 0.019*** | 0.020*** |
|  |  | MR |  | 0.004 |
| Stiff portion | Female | S | 0.022* | 0.027** |
|  |  | MR |  | 0.032** |
|  | Male | S | 0.034*** | 0.035*** |
|  |  | MR |  | 0.014 |

** p<0.01, * p < 0.05
