# Supplementary material for: Morphofunctional characteristics of flight-related traits in deltamethrin-resistant and susceptible Triatoma infestans (Klug, 1834) of the Argentinean Chaco
Source: Parasit Vectors. 2025 Mar 6;18:92. doi: 10.1186/s13071-025-06678-2 (PMC11883966; doi:10.1186/s13071-025-06678-2)
Supplement: Supplementary file 3 — Additional file 3. Mahalanobis distances across phenotypic toxicological groups of Triatoma infestans for the shape of the flight-related traits: forewing, head and membranous and stiff portions of the forewing. Toxicological group names are as in Table 1. [file 13071_2025_6678_MOESM3_ESM.docx]

Additional file 3

| **Measurement** | **Sex** | **Toxicological group**  **MR HR** | | |
| --- | --- | --- | --- | --- |
| Forewing | Female | S | 3.086*** | 2.752*** |
|  |  | MR |  | 1.928*** |
|  | Male | S | 2.622*** | 3.448*** |
|  |  | MR |  | 2.149*** |
| Head | Female | S | 1.219** | 1.442*** |
|  |  | MR |  | 1.563*** |
|  | Male | S | 0.755* | 2.596*** |
|  |  | MR |  | 2.895*** |
| Membranous portion | Female | S | 3.169*** | 4.485*** |
|  |  | MR |  | 2.510** |
|  | Male | S | 2.994*** | 3.229*** |
|  |  | MR |  | 0.517 |
| Stiff portion | Female | S | 2.887*** | 4.060*** |
|  |  | MR |  | 2.753*** |
|  | Male | S | 2.953*** | 3.538*** |
|  |  | MR |  | 2.059** |

** p<0.01, * p < 0.05
